# Characterisation of Dendritic Cells Arising from Progenitors Endogenous to Murine Spleen

**DOI:** 10.1371/journal.pone.0088311

**Published:** 2014-02-14

**Authors:** Sawang Petvises, Helen C. O’Neill

**Affiliations:** Division of Biomedical Science, Research School of Biology, The Australian National University, Canberra, ACT, Australia; Oklahoma Medical Research Foundation, United States of America

## Abstract

Heterogeneity amongst dendritic cell (DC) subsets leads to a spectrum of immune response capacity against pathogens. Several DC subsets in spleen have been described which differ in terms of phenotype and function. We have previously reported a distinct population of CD11c^lo^CD11b^hi^MHC-II^−^CD8^−^ dendritic-like “L-DC” in murine spleen, which can also be generated in splenic stromal longterm cultures. Here, the ontogeny of L-DC development in perinatal mice has been compared with other known splenic DC subsets. Flow cytometric analysis has revealed the presence of L-DC at embryonic age (E)18.5 spleen, while plasmacytoid (p)DC and conventional (c)DC appear at 2 and 4 days following birth. Co-cultures of E18.5 spleen above splenic stroma also showed production of only L-DC, while spleen cells from D0 through D5 neonates showed production of both L-DC and cDC-like cells. Addition of an M-CSFR inhibitor to co-cultures revealed that while the development of cDC-like cells depended on M-CSF, many L-DC developed independently of M-CSF. Furthermore, purified hematopoietic stem cells (HSC) and multipotential progenitors (MPP) isolated from neonatal D1 spleen are capable of developing into L-DC in co-cultures. These studies reveal a lineage of dendritic-like cells developing in the spleen microenvironment, and which appear to arise from endogenous progenitors laid down in spleen during embryogenesis.

## Introduction

Hematopoiesis in fetal spleen occurs at around embryonic day (E)14.5. Hematopoietic stem cells (HSC) in fetal spleen have limited proliferative ability, and a small number of HSC and immediate progenitors also emigrate from fetal liver to spleen [1]. Spleen hematopoiesis is believed to be restricted to production of erythyrocytes with minor myeloid lineage development, particularly dendritic cells (DC) [2]. However, the development of DC during embryogenesis and perinatal life has not been fully investigated.

Several studies have now demonstrated the presence of HSC in steady-state adult spleen, albeit in low numbers [1], [3], [4]. Osteoblastic and vascular niches are sites of HSC maintenance, proliferation and differentiation in bone marrow (BM), but the splenic niche for HSC has not been well defined [5]. The spleen contains only vascular niches and no osteoblastic sites, so the maintenance and differentiation of HSC in the spleen microenvironment may be mechanistically different to that of BM. Indeed, while splenic stromal cells have been found to express signaling molecules similar to those described in BM hematopoietic niches [6], it has been determined that HSC cannot be maintained in E14.5 fetal spleen organ cultures [7]. Here we describe a murine spleen stromal cell line derived from a 6-day old (D6) mouse spleen which does support hematopoiesis, but only of dendritic-like cells [8], [9], [10].

In the steady-state, adult spleen contains several commonly known DC subsets including conventional (c)DC, plasmacytoid (p)DC and monocyte-derived DC whose development relies on the continuous supply of immediate DC precursors seeding through blood from BM to spleen, where they complete their development in the spleen microenvironment [11]. While these DC subsets are now well described in the literature, they are readily distinguishable from a smaller subset of dendritic-like cells which we have described: a CD11b^hi^CD11c^lo^MHC-II^−^ splenic subset called “L-DC” which are also F4/80^+^Ly6C^−^4-1BBL^lo^ [12], [13] (also unpublished data). These cells are distinct in that they induce CD8^+^ T cell responses, but do not activate CD4^+^ T cells. Previous studies had shown that long-term cultures (LTC) of neonatal spleen maintained production of similar dendritic-like cells called “LTC-DC” over years, suggesting that they may be derived from self-renewing progenitors [14], [15], [16]. Cloned splenic stroma derived from LTC have since been shown to support development of equivalent cells called “L-DC” from overlaid lineage-depleted (Lin^−^) BM or purified HSC [8], [17], [18]. When cells produced in co-cultures or LTC were collected and sorted, the CD11b^−^CD11c^−^ subset was found to contain L-DC progenitors and could re-seed stroma for L-DC production [8], [9]. The CD11c^+^CD11b^+^ subset could not however, and overlaid cells died without differentiating further. In a previous study it was also confirmed that L-DC do not derive from a monocyte or myeloid precursor since CD11b^+^MHC-II^−^ cells from spleen did not seed stromal co-cultures for hematopoiesis [19].

The *in vivo* equivalent of L-DC is now characterised in adult spleen [12], and L-DC are distinct from splenic cDC and pDC in terms of their phenotype, their high endocytic capacity, and their capacity for cross-presentation of antigen to CD8^+^ T cells [13], [18]. L-DC are also distinct from monocytes, and in particular a CD11b^lo^CD11c^lo^MHC-II^−^ subset of small (FSC^lo^) spleen cells which others have classified as “residential monocytes” [20], [21] and which we tentatively classified as “DC precursors” [12], since they reflect a heterogeneous population of CD11c^+^ cells. L-DC are distinct from this subset in that they have a distinct FSC^hi^ profile, are highly endocytic and can cross present antigen which the CD11b^lo^CD11c^lo^MHC-II^−^ cells cannot do [12].

The possibility that spleen maintains endogenous progenitors of L-DC is of immense biological interest in terms of tissue-specific hematopoiesis, and the possible production of spleen-specific antigen presenting cells having tissue-specific function. Indeed, their development may be attributable to the development of stromal niches during the neonatal period, and the influx of hematopoietic progenitors into tissues during embryogenesis. The ontogeny of L-DC development in perinatal spleen has been determined here in relation to other known cDC and pDC subsets. The presence of L-DC progenitors in neonatal spleen was also tested by overlaying cells in co-cultures above 5G3 stroma. The importance of Flt3L, GM-CSF and M-CSF for L-DC development in spleen has also been considered in light of evidence for the role of these factors in the development of cDC and pDC.

## Materials and Methods

### Ethics Statement on Animals

Animal housing, handling and experimentation was approved by the Animal Experimentation Ethics Committee (Australian National University, Canberra, Australia) and experiments performed under protocol number A2013/11. Specific pathogen free female C57BL/6J mice were bred at the John Curtin School of Medical Research (JCSMR) (Canberra, Australia). Animals were sacrificed by cervical dislocation.

### Antibodies

Purified antibodies specific for CD3ε (145–2C11), CD4 (L3T4), CD5 (53–7.3), CD8 (53–6.7), B220 (RA3–6B2), Gr-1 (RB6–8C5), Ter119 (Ter-119), CD16/32 (93) and Mac-1 (M1/70) were obtained from eBioscience (San Diego, CA, USA). Fluorochrome-conjugated antibodies specific for CD11c (N418), CD11b (M1/70), and streptavidin-APC-Cy7 were obtained from eBioscience. Fluorochrome-conjugated antibodies specific for CD3 (145–2C11), CD8 (53–6.7), CD19 (MB19–1), Gr-1 (RB6–8C5), Ter119 (Ter-119), B220 (RA3–6B2), Ly6C (AL-21), Ly6G (1A8), MHC-II (AF6–120.1), F4/80 (C1: A3–1), c-kit (2B8), Sca1 (E13–161.7), Flt3 (A2F10), CD150 (TC15–12F12.2), 4–1BBL (TKS-1), streptavidin-PE-Cy7, streptavidin-PE and streptavidin-FITC were obtained from Biolegend (San Gabriel, CA, USA). Isotype control antibodies including Rat IgG_2a_ (R35–95), Rat IgG_2b_ (RTK4530), Rat IgG_2b_ (eB149/10H5), Mouse IgG_2a_ (eBM2a) and Hamster IgG (eBio299Arm) were obtained from eBioscience. All antibodies were titrated prior to use to determine concentration giving minimum saturation binding.

### Cell Culture and Reagents

Cells were cultured in DMEM supplemented with 4g/L D-glucose, 6mg/L folic acid, 36mg/L L-asparagine, 116mg/L L-arginine, to which was added 10% FCS, 10mM HEPES, 2mM L-glutamine, 100U/L penicillin, 100ug/L streptomycin and 5×10^−5^M 2-mercaptoethanol. The splenic stromal cell line 5G3 [22], [23] was passaged every 4 days by scraping and transferring non-adherent cells to a new flask. Cells were maintained in 5% CO_2_ in 95% humidity at 37°C.

### Preparation of T/B Depleted Spleen Cells

A suspension of T and B cell depleted adult spleen cells was used for DC subset analysis and co-culture above 5G3 stroma. Biotinylated antibodies specific for CD19 (eBio1D3: mouse IgG_2a_) (eBioscience), Thy 1.2 (30-H12: rat IgG_2b_) (Becton Dickinson: San Diego, CA, USA) and Ter-119 (TER-119: rat IgG_2b_) (Biolegend) were used to remove red blood cells as well as T and B lineage cells. For these experiments, antibodies to Thy1.2, CD19, and Ter119 were absorbed to 10^7^ cells followed by incubation on ice for 20 minutes. The cell suspension was then washed twice with labeling buffer by centrifugation at 300 g and 4°C for 5 minutes. The supernatant was discarded completely and cells resuspended in 20 ul of MACS® anti-biotin microbeads (Miltenyi Biotech: Gladbach, Germany). Cells were further incubated on ice for 20 minutes. Following incubation, cells were washed twice with 10 ml labeling buffer by centrifugation at 300 g and 4°C for 5 minutes. The supernatant was completely decanted and cell pellets resuspended in 500 ul labeling buffer. Cell suspension was transferred to a MACS® MS column for cell separation.

### Co-culture of Spleen Cells Over 5G3 Stroma

For establishment of co-cultures, spleen cells (10^4–5^ cells/ml) were overlaid on to near-confluent 5G3 stroma in replicate 25 cm^2^ flasks (5 ml). Medium change was performed every 3–4 days by discarding 2.5 ml medium and replacement with 2.5 ml fresh-warmed complete medium. Non-adherent cells produced in co-cultures were collected at days 14, 21 and 28 for analysis of cell subsets produced. In the M-CSFR inhibition study, 10^4–5^ cells/ml sorted longterm (LT)-HSC and multipotent progenitors (MPP) from one-day old spleen of C57BL/6J mice were overlaid on to 5G3 stroma in the presence and absence of 10 nM GW2580, an M-CSFR inhibitor (BioVision, CA, USA). Non-adherent cells were then collected at days 14, 21, and 28 for analysis of cells produced.

### Cytokine-induced Production of DC

Whole BM or spleen cells were resuspended at a final concentration of 1×10^6^ cell/ml in RPMI 1640 medium supplemented with 10% FCS, 5×10^−4^ M 2-mercaptoethanol, 10 mM 4-(2-hydroxyethyl)-1-piperazineethanesulfonic acid (HEPES), 100 U/ml penicillin, 100 ug/ml streptomycin (sRPMI 1640). Flt3L (Genzyme: Cambridge, MA, USA) was added to cultures at a final concentration of 200 ng/ml. GM-CSF (Genzyme) was added at a final concentration of 10 ng/ml, and IL-4 (Genzyme) at 0.3 ng/ml. Cells were maintained at 37°C, 5% CO_2_ in air and 95% humidity. Medium was changed by replacing half of aged medium with fresh warm sRPMI 1640 medium supplemented with cytokines as described above. GM-CSF/IL-4 cultures were collected by vigorously shaking the flask with removal of supernatant at 7 days, while cells in Flt3L cultures were collected at 9 days for analysis of surface marker expression by antibody staining and flow cytometry.

### Isolation of Hematopoietic Progenitors from Neonatal Spleen

Spleen cells from one-day old mice were stained with antibodies for delineation of progenitors for sorting. A cocktail of antibodies was used to sort Lin (CD3, CD4, CD5, CD8, B220, Gr-1, CD11b, Ter118, CD11c) ^−^ cells. LT-HSC were sorted as Lin^−^Sca-1^+^c-kit^+^Flt3^−^CD150^+^ cells [21]; MPP as Lin^−^Sca-1^+^c-kit^+^Flt3^+^CD150^−^ [24]; sorting was performed on a FACSAria II flow cytometer (Becton Dickinson), and sorted cells reanalysed to check purity. Isotype control antibodies were used to set gates and propidium iodide (PI: 1 ug/mL) staining of cells was used for dead cell discrimination. Sorted cells were washed twice and overlaid on to 5G3 stroma for assessment of cell production.

### Analysis of Cells Produced in Co-cultures

Cells collected from co-cultures at each time point were stained with antibodies specific to dendritic and myeloid cells. Briefly, 10^5–6^ cells were firstly incubated with purified CD16/32 antibody for 15 min to block surface Fc receptors. After incubation, cells were washed with DMEM/1%FCS/0.1%NaN_3_ and then stained with primary antibodies specific for CD11c, CD11b, CD8, B220 and MHC-II for 20 min on ice. In some experiments, Ly6C, Ly6G, F4/80 and 4-1BBL were used to detect specific subsets in co-cultures. Secondary antibodies were added to the stained cells after a washing step, and further incubated for 20 min on ice. Stained cells were finally washed twice and resuspended in 70 ul sDMEM/1%FCS/0.1%NaN_3_ in FACS cluster tubes. Cells were stained with PI (1 ug/ml) for live cell discrimination. Cell acquisition was performed using a LSRII flow cytometer (Becton Dickinson). Between 5×10^4^ and 10^6^ events were collected for each sample. Gates were set to delineate cell subsets using isotype control antibodies and fluorescence minus one controls. Cell subset analysis was performed using BD FACSDiva Software (Becton Dickinson) and FlowJo Software (Tristar; Phoenix, Arizona, USA).

## Results

### L-DC Appear Earlier than cDC and pDC in Neonatal Spleen

**Figure 1:**
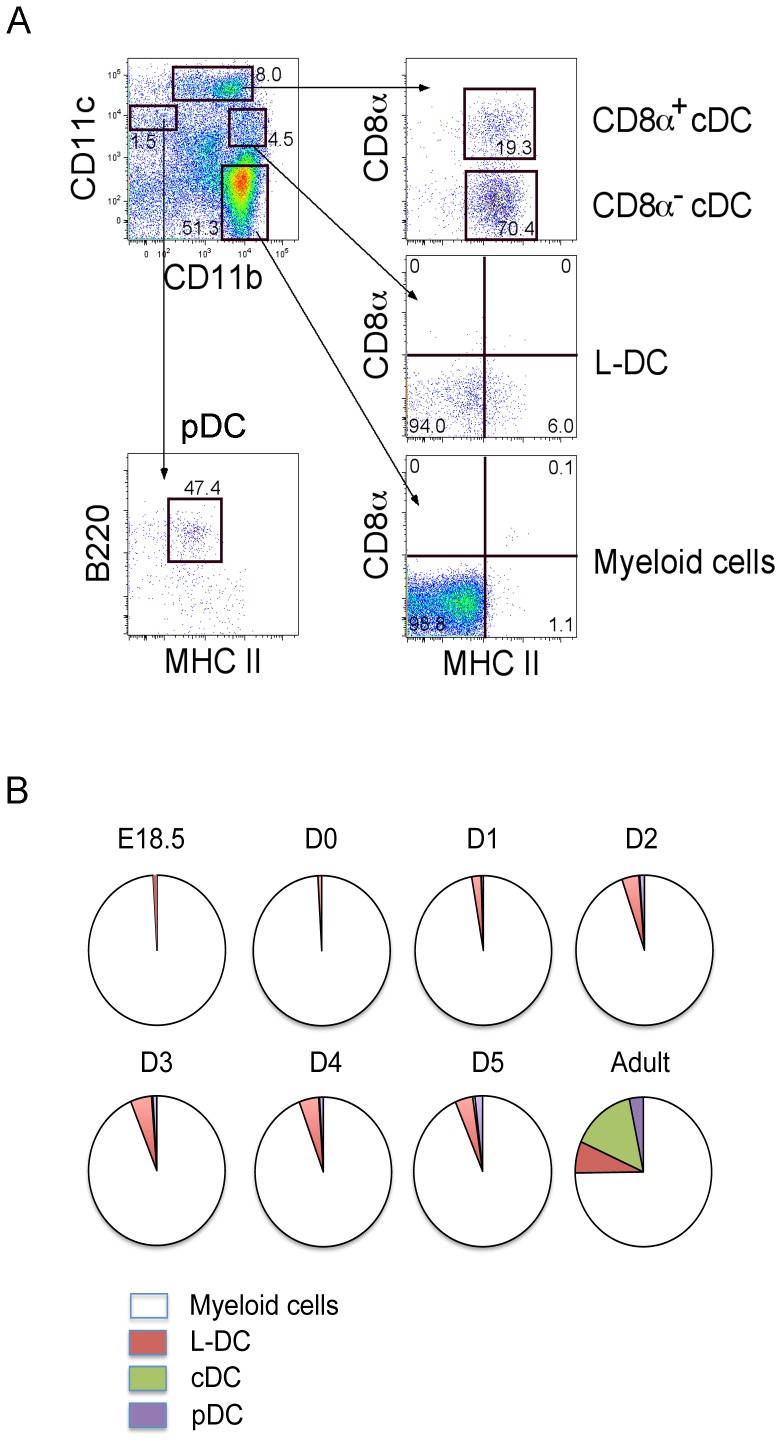
Characterization of splenic DC subsets. (**A**) Perinatal and adult spleen cells from C5BL/6J mice were stained with antibodies to distinguish DC subsets flow cytometrically. The procedure for gating subsets in one adult mouse is shown by example. Propidium iodide (PI) staining was used to discriminate dead cells. Gates were set on bivariate plots using isotype control antibodies and numbers on gates reflect % positive cells. CD11b and CD11c staining was used to identify CD11b^hi^CD11c^−^, CD11b^hi^CD11c^lo^, CD11b^+^CD11c^hi^ and CD11b^−^CD11c^+^ subsets. Further staining for CD8, MHC-II and B220 was used to distinguish CD8^+^ cDC and CD8^−^ cDC, L-DC, myeloid cells, and pDC. (**B**) The proportional representation of myeloid and DC subsets amongst the total CD11b^+^ and/or CD11c^+^ dendritic/myeloid population is shown. Data were derived using average values obtained for 2 mice. E =  embryonic; D =  day post birth.

Perinatal spleen cells were assessed by antibody staining and flow cytometric analysis in order to identify DC subsets in comparison with adult spleen. Analysis involved initial gating of cell subsets differing in expression of CD11b, CD11c, MHC-II, B220 and CD8. The CD11b^lo^CD11c^hi^MHC-II^+^ subset was divided on the basis of CD8 expression to give CD8^+^ and CD8^−^ cDC subsets. The CD11b^hi^CD11c^lo^MHC-II^−^CD8^−^ subset was delineated as L-DC [12]. CD11b^+^CD11c^−^MHC-II^−^CD8^−^ cells were distinguished as myeloid cells. The CD11b^−^CD11c^+^ subset was further distinguished as a CD11b^−^CD11c^+^B220^+^MHC-II^+^pDC (Fig. 1A).

Analysis of DC subsets appearing in perinatal spleens revealed L-DC appearing as early as E18.5, while pDC and cDC appeared at D2 and D5, respectively. CD11b^+^CD11c^−^CD8^−^MHC-II^−^ myeloid cells predominated during early spleen development from E18.5 to adult. One explanation for the presence of L-DC but not cDC and pDC in E18.5 spleen, is that L-DC in perinatal spleen might derive from endogenous progenitors laid down in spleen during embryogenesis (Fig. 1B). Indeed, L-DC might develop from yolk sac-derived HSC which enter spleen during embryogenesis. The cDC and pDC subsets are known to derive from BM precursors which enter spleen for further development [25].

### Pre-natal Spleen Contains only L-DC Progenitors

**Figure 2:**
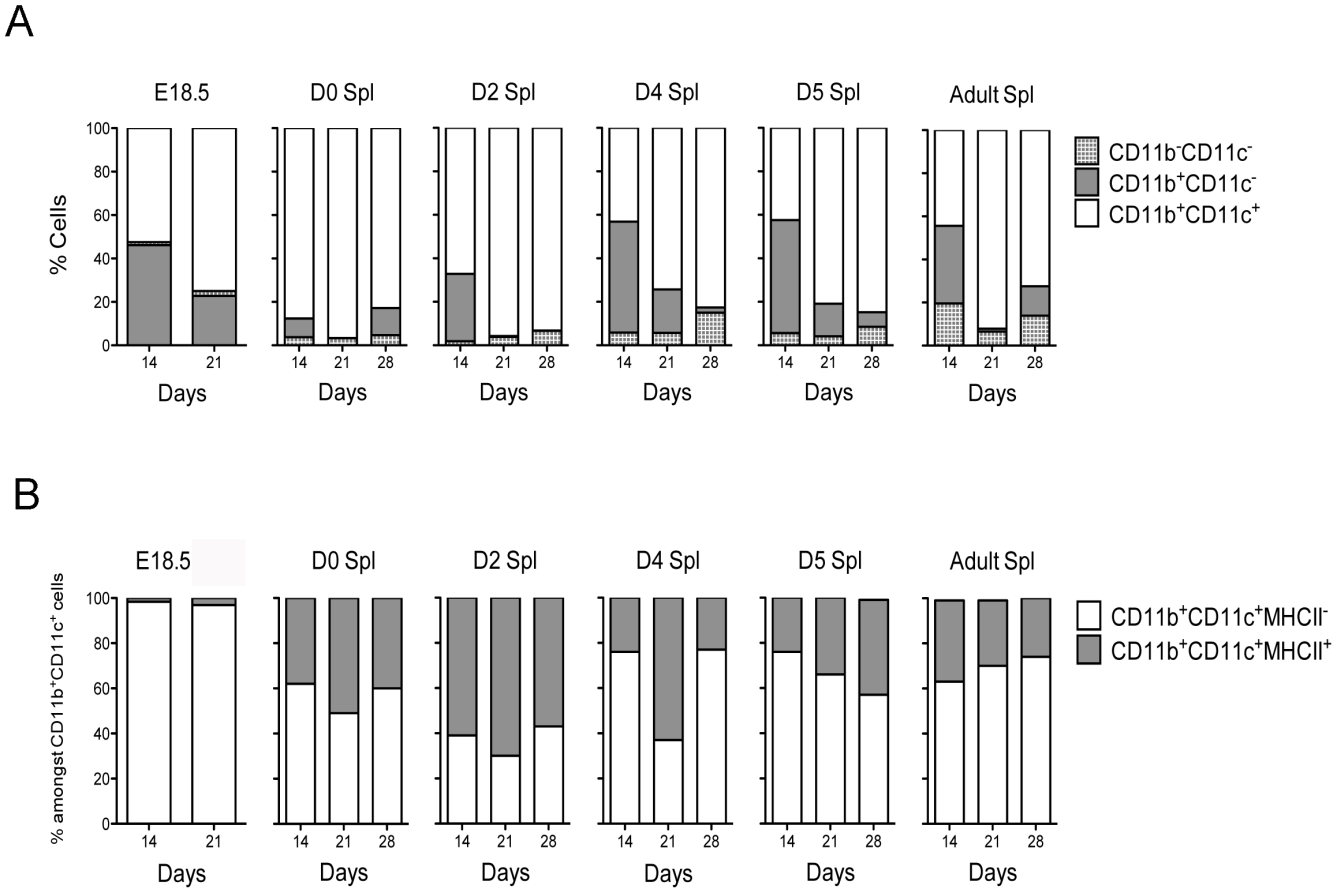
Production of dendritic-like cells in co-cultures established with perinatal spleen. (**A**) Co-cultures of E18.5, D0, D2, D4, D5 and adult spleen cells were established over 5G3 stroma. Cell production was assessed after 14, 21 and 28 days using antibody staining and multicolor flow cytometry. Gates were set on bivariate plots using isotype control antibodies and numbers on gates reflect % positive cells. Production of CD11c and CD11b distinct subsets was calculated in terms of the relative proportion of each. (**B**) Production of CD11b^hi^CD11c^lo^MHC-II^−^ L-DC, and CD11b^hi^CD11c^hi^MHC-II^+^ cDC-like cells was calculated in terms of the relative proportion amongst the total CD11c^+^ and/or CD11b^+^ cells.

The presence of progenitors of DC subsets was then investigated in perinatal spleen. Co-cultures were established by overlay of red blood cell lysed perinatal spleen cells on 5G3 stroma with flow cytometric assessment of the production of L-DC and cDC-like cells. The small tissue size precluded further purification of spleen cell suspensions. Gates were used to delineate DC (CD11b^+^CD11c^+^), myeloid cells (CD11b^+^CD11c^−^) and progenitors (CD11b^−^CD11c^−^). DC could be distinguished as two subsets of L-DC (CD11b^hi^CD11c^+^MHC-II^−^) and cDC-like cells (CD11b^+^CD11c^hi^MHC-II^+^). Co-cultures of E18.5 spleen were shown to maintain 50–80% of cells as DC, with 20–45% myeloid cells varying slightly over time. Co-cultures of D0, D2, D4, D5 and adult spleen also maintained a majority of DC with variable numbers of myeloid cells and increasing numbers of progenitors (Fig. 2A). Data are presented in terms of % subset representation amongst total dendritic and myeloid cells rather then absolute cell numbers since spleen size, and cell composition varies in spleens of different perinatal age. In terms of DC subsets produced, E18.5 spleen co-cultures produced only L-DC, while a subpopulation of cDC-like cells was observed along with L-DC in D0, D2, D4, D5, D7 and adult spleen co-cultures (Fig. 2B). Also of note is a biphasic peak in appearance of L-DC in cultures established from E18.5 and D4 spleen. It is tempting to propose that the higher productivity of L-DC at these times reflects cell development from primitive HSC (E18.5), and then later from definitive HSC (D4). These data also indicate the presence of only L-DC progenitors in pre-natal E18.5 spleen, with almost no evidence of cDC progenitors (Fig. 2B). After birth, precursors of cDC-like cells appeared in spleen.

### *In vitro* Induction of DC Using Flt3L and GM-CSF

**Figure 3:**
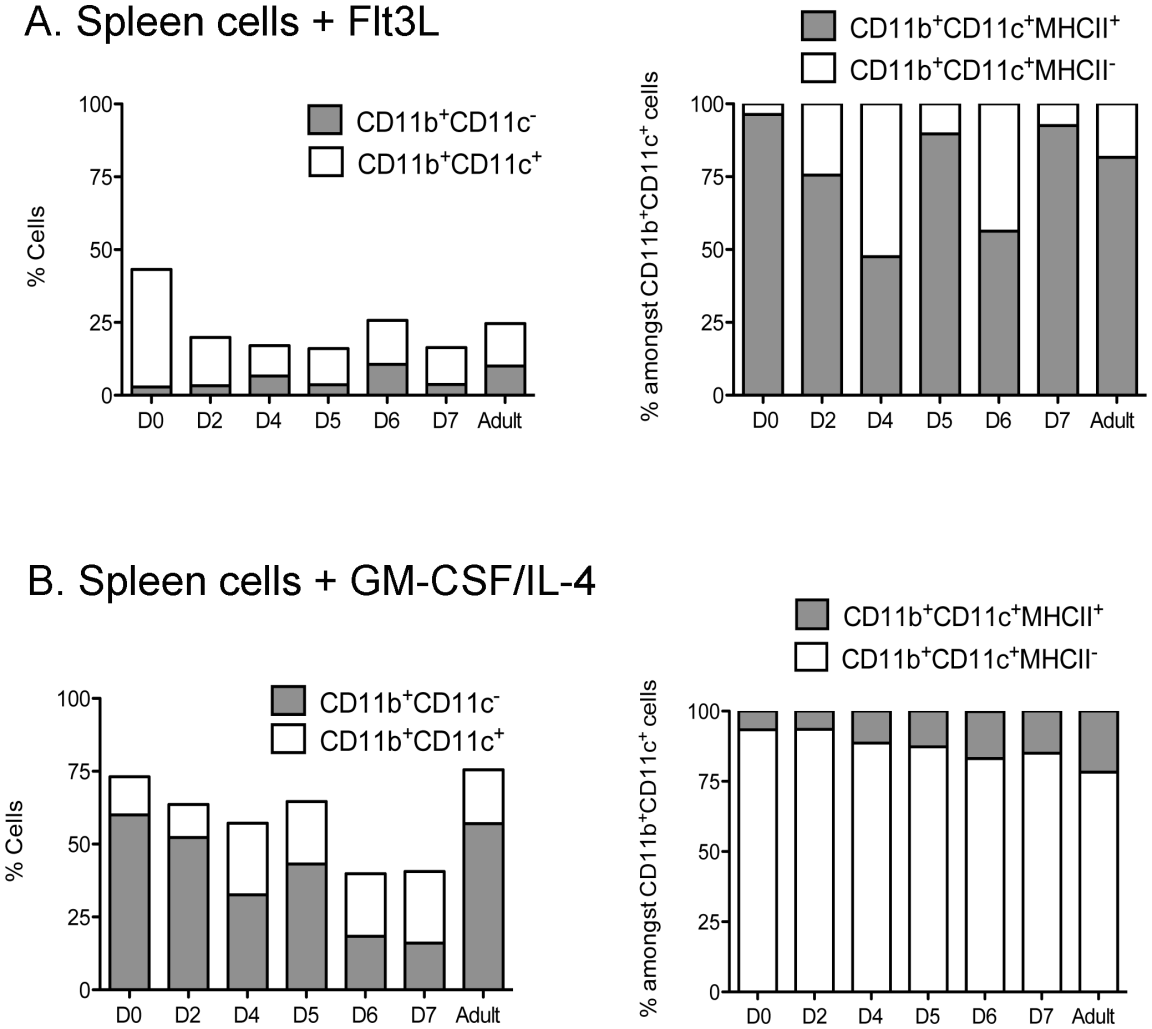
Production of DC in Flt3L and GM-CSF/IL-4 supplemented cultures of spleen. (**A**) D0, D2, D4, D5, D6, D7 and adult spleen cells were cultured with Flt3L or GM-CSF/IL-4. Cell production was assessed using antibody staining and multicolor flow cytometry. Gates were set on bivariate plots using isotype control antibodies, and numbers in gates reflect % positive cells. Production of CD11b^+^CD11c^−^ and CD11b^+^CD11c^+^ cells was calculated in terms of proportion of each subset amongst all CD11c^+^ and/or CD11b^+^ cells. Representation of CD11b^+^CD11c^+^MHC-II^−^ (L-DC) and CD11b^+^CD11c^+^MHC-II^+^ (cDC-like) cells was calculated in terms of proportion amongst CD11b^+^CD11c^+^ cells. (**A**) Proportion of cells induced by Flt3L. (**B**) Proportion of cells induced by GM-CSF/IL-4.

**Figure 4:**
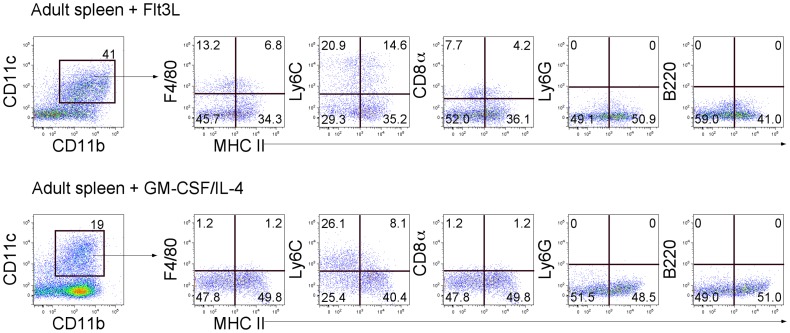
Phenotype of DC derived from adult spleen cells cultured with Flt3L or GM-CSF/IL-4. Spleen cell cultures were supplemented cultures with Flt3L and GM-CSF/IL-4 were analysed for production of CD11c^+^CD11b^+^ DC. These subsets were further distinguished by expression of MHC-II, F4/80, Ly6C, CD8, Ly6G and B220.

In order to investigate DC development more fully, the presence of DC precursors responsive to Flt3L and GM-CSF/IL-4 was investigated over the perinatal period in *in vitro* cultures of neonatal spleens, since these factors are known to regulate DC development from both *in vivo* and *in vitro* studies [26], [27]. Spleen cells from D0, D2, D4, D5 and D7 mice were cultured in the presence of Flt3L, and cell production compared with cultures from adult mice. Since all cultures produced equivalent numbers of cells under the culture conditions imposed (data not shown), cell production was compared in terms of % subset representation. The production of DC as CD11b^+^CD11c^+^ cells was higher than the production of CD11b^+^CD11c^−^ myeloid cells across this period (Fig. 3A). Cells were collected for detection of CD11b^+^CD11c^+^MHC-II^−^ L-DC and CD11b^+^CD11c^+^MHC-II^+^ cDC-like subsets. Flt3L cultures maintained levels of 50–97% of MHC-II^+^ cDC-like cells, with a lower 3–50% population of MHC-II^−^ L-DC-like cells across all ages of mice (Fig. 3A). Further staining revealed production of a minor subset of CD11b^+^CD11c^+^MHC-II^−^cells which express F4/80 consistent with L-DC production (Fig. 4). However, not all MHC-II^−^ cells are L-DC since 40% express Ly6C, which is a marker of monocyte-like cells. Flt3L cultures therefore produce a mixture of cell types including a minor population of CD8^+^ cDC as reported previously (Fig. 4) [27]. However, no granulocytes (Ly6G) or plasmacytoid DC (B220) were detected. Therefore, Flt3L cultures are more conducive to the development of cDC than L-DC, and precursors are present from D0 to D7 embryos, and in adults. The presence of precursors contrasts with the absence of detectable numbers of cDC in spleens until D5, and then in very low numbers (Fig. 1B).

Similar spleen cultures supplemented with GM-CSF and IL-4 also produced equally high numbers of cells so that cell production was compared in terms of proportional subset representation. Cultures maintained 20–60% populations of CD11b^+^CD11c^−^ myeloid cells with lower representation of CD11b^+^CD11c^+^ DC particularly from D0 to D5 (Fig. 3B). The proportion of CD11b^+^CD11c^+^MHC-II^−^ cells representing L-DC amongst all CD11b^+^ and/or CD11c^+^ myeloid cells was >80% in all cultures established with D0 to D7 spleens, suggesting that GM-CSF and IL-4 promote the development of cells of similar CD11b^+^CD11c^+^MHC-II^−^ phenotype to L-DC (Fig. 3B). However, further staining of these CD11b^+^CD11c^+^MHC-II^−^ cells confirmed that they were not L-DC since they did not express F4/80, a known L-DC marker (Fig. 4). Some CD11b^+^CD11c^+^MHC-II^−^ cells expressed Ly6C which also serves to discount them as L-DC, while cells were also negative for CD8, expression of Ly6C on a subset is consistent with production of a CD11c^lo^ monocyte-like population.

It seems unlikely therefore that GM-CSF/IL-4 supports the development of L-DC but may support development of cDC-like cells and other immature dendritic-like cells (Fig. 4). A subset of CD11b^+^CD11c^+^MHC-II^−^ cells produced in Flt3L-supplemented culture, are more reflective of L-DC grown in 5G3 co-cultures, in that they express F4/80, but not Ly6C [13].

### M-CSF Directs DC Differentiation in Stromal Co-cultures

**Figure 5:**
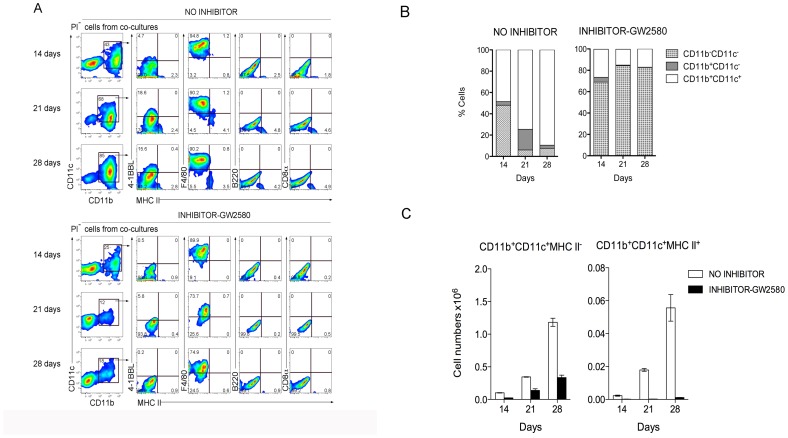
An M-CSFR inhibitor inhibits development of cDC-like cells but not L-DC in neonatal spleen co-cultures. One-day old splenocytes of C57BL/6J mice were co-cultured with 5G3 stroma in the presence or absence of 10ng/ml GW2580, an inhibitor of M-CSFR, added to cultures at medium change every 3–4 days. (**A**) Cell production was analysed at 14, 21 and 28 days, by staining cells for CD11c, CD11b, MHC-II, 4-1BBL, F4/80 and CD8, followed by flow cytometric analysis. Propidium iodide staining was used to delineate live (PI^−^) cells, which were then gated on CD11c and CD11b using isotype controls to set gates. L-DC were then distinguished from cDC-like cells on the basis of expression of 4-1BBL, F4/80 and absence of MHC-II expression. Results from one representative culture out of 3 replicates is shown. (**B**) Co-cultures were analysed over time for production of CD11b^+^ cells which differed in expression of CD11c, as well as the CD11b^−^CD11c^−^ progenitor-containing subset. (**C**) Relative production of CD11b^+^CD11c^+^MHC-II^−^ L-DC and CD11b^+^CD11c^+^MHC-II^+^ cDC-like cells was monitored over time in terms of absolute number of cells produced. Values represent mean ± S.E. of three replicate cultures established from individual mice.

A role for M-CSF in DC development in 5G3 co-cultures was also investigated, since previous studies have shown high levels of M-CSF produced by the 5G3 stromal line [6]. The GW2580 inhibitor of M-CSFR was added into co-cultures established from one-day old spleens of C57BL/6J mice and cell production compared with controls. The inhibitor was replenished at medium change every 2 days to maintain the concentration of inhibitor over 28 days. Cells were collected for analysis of the production of L-DC and cDC-like cells at 14, 21 and 28 days. Production of cells was assessed flow cytometrically by gating CD11b^+^CD11c^+^ DC, followed by analysis of expression of MHC-II, 4-1BBL, F4/80, and CD8 to delineate DC subsets. Cultures produced 2 main populations of cells, a CD11b^+^CD11c^+^ DC population, and a CD11b^−^CD11c^−^ progenitor-containing population (Fig. 5A). The size of the CD11b^−^CD11c^−^ progenitor/precursor-containing population was notably larger in the presence of inhibitor, comprising up to 82% of cells across 28 days of co-culture, compared with ∼15% of all cells in control co-cultures (Fig. 5B). The small subset of CD11b^+^CD11c^+^MHC-II^+^ cDC-like cells (3–5%) produced in “no inhibitor” controls was distinguishable from the major population of CD11b^+^CD11c^+^MHC-II^−^ L-DC also confirmed by their expression of F4/80 and 4-1BBL (Fig. 5A). In contrast, almost no CD11b^+^CD11c^+^MHC-II^+^ cDC-like cells were maintained in co-cultures containing the GW2580 inhibitor (Fig. 5A). Overall, in “no inhibitor” control cultures, ∼25 times more L-DC were produced than cDC-like cells (Fig. 5C). The effect of the inhibitor was to reduce the production of L-DC by ∼3 fold, while the production of cDC-like cells was reduced by at least 50-fold (Fig. 5C). These data suggest that M-CSF is important for the development of only some L-DC, but completely regulates the production of cDC-like cells and progenitors (Fig. 5B). Since the size of the progenitor-containing subset increased dramatically in the presence of GW2580, it appears that M-CSF is an important regulator not only of the development of cDC-like cells, but also of progenitor development. Since so few cDC are produced in control “no inhibitor” cultures, it appears that M-CSF may act as a negative regulator of progenitor proliferation, while perhaps supporting progenitor differentiation to c-DC. Upon inhibition of M-CSF signaling there is a disproportionate increase in the progenitor pool, in relation to the decrease in production of cDC-like cells.

### Spleen Contains LT-HSC which Produce Only L-DC in Stromal Co-cultures

**Figure 6:**
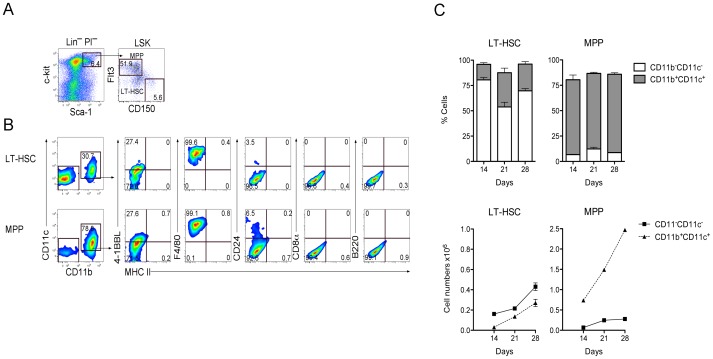
LT-HSC in neonatal spleen contribute to DC production in stromal co-cultures. (**A**) Spleen cells isolated from one-day old C5BL/6J mice were stained with antibodies to distinguish hematopoietic progenitors flow cytometrically. A lineage cocktail of antibodies (CD3, CD4, CD5, CD8, B220, Gr-1, CD11b, Ter119, CD11c) was used to exclude mature Lin^+^ cells. Staining with Propidium iodide (PI:1ugm/ml) was used to delineate PI^−^ live cells. Sca-1 and c-kit staining was used to identify the Lin^−^c-kit^+^Sca-1^+^(LSK) subset. Staining with Flt3 and CD150 was used to distinguish and sort LT-HSC and MPP, which were overlaid in co-cultures above 5G3 stroma. (**B**) Non-adherent cells produced in co-cultures were stained with antibodies to detect a CD11b^+^CD11c^+^ population of dendritic-like cells. These were tested for expression of MHC-II, 4-1BBL, F4/80, CD24, CD8, and B220 to delineate L-DC and cDC-like subsets. Data show cell production at 21 days for one of three replicate cultures established from different mice. Gates were set on bivariate plots using isotype control antibodies and numbers on gates reflect % positive cells. (**C**) Production of CD11b^−^CD11c^−^ progenitors and CD11b^+^CD11c^+^ dendritic-like cells was calculated in terms of proportion of each subset and number of cells of each type produced. Graphs show mean±S.E. for triplicate co-cultures.

Since HSC from BM have previously been shown to represent a source of L-DC progenitors [17], [18], we questioned whether neonatal spleens contained HSC. T/B depleted spleen cells were prepared from one-day old mice for analysis and sorting of HSC and progenitor subsets by antibody staining and flow cytometry. Live (PI^−^) cells were gated and mature lineage (Lin^+^) cells excluded. Sca-1 versus c-kit expression was used to delineate the Lin^−^Sca-1^+^c-kit^+^ (LSK) subset (Fig. 6A). Differential expression of Flt3 and CD150 was then used to divide the LSK subset further. LT-HSC were identified as Flt3^−^CD150^+^Lin^−^Sca-1^+^c-Kit^+^ cells, and MPP as Flt3^+^CD150^−^Lin^−^Sca-1^+^c-Kit^+^ cells, based on previous studies (Fig. 6A) [24], [28]. In order to determine the production of DC, sorted LT-HSC and multipotential progenitors (MPP) were overlaid on to 5G3 stroma, and cells collected for analysis at 14, 21 and 28 days. Gating on the CD11b versus CD11c plot was used to delineate 2 populations evident as DC (CD11b^+^CD11c^+^), and a progenitor-rich (CD11b^−^CD11c^−^) population. The expression of MHC-II, B220, CD8, F4/80 and 4-1BBL also helped to distinguish L-DC (Fig. 6B).

**Figure 7:**
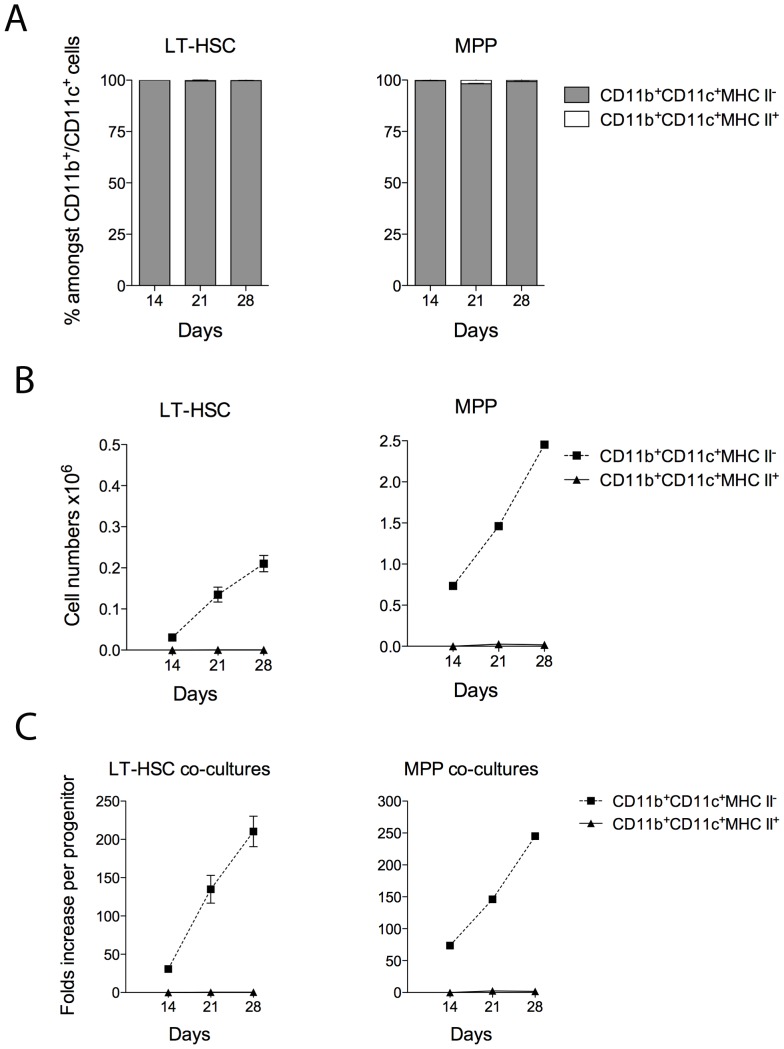
LT-HSC co-cultures produce only L-DC. Sorted LT-HSC and MPP were used to establish co-cultures above 5G3 stroma as described in Fig. 6. Non-adherent cells were collected and stained with antibody to detect subsets of dendritic-like cells. The production of CD11b^+^CD11c^+^MHC-II^−^ L-DC and CD11b^+^CD11c^+^MHC-II^+^ cDC-like cells was calculated in terms of (**A**) proportion of each subset amongst CD11b^+^ and/or CD11c^+^ cells, (**B**) the each cell type produced, and (**C**) relative yield of each cell type in relation to number of progenitors used to establish co-cultures. Graphs show mean±S.E. for triplicate co-cultures.

LT-HSC co-cultures maintained >50% CD11b^−^CD11c^−^ progenitor-containing subset, with ≤30% CD11b^+^CD11c^+^ DC produced over 28 days (Figures 6B & 6C). In contrast, MPP co-cultures maintained a <15% CD11b^−^CD11c^−^ subset and >65% myeloid cells over this period. In terms of cell numbers, MPP co-cultures yielded higher numbers of DC than did LT-HSC cocultures, although lower numbers of progenitors (Fig. 6C). Both LT-HSC and MPP co-cultures produced only L-DC, consistent with a model for direct differentiation of L-DC from self-renewing progenitors present in these 2 subsets (Fig. 7A and 7B). Due to lower available numbers of LT-HSC in bone marrow, co-cultures were established with only 10^3^ cells, compared with 10^4^ for MPP. However, these two progenitor subsets had the same capacity for production of L-DC, yielding a similar increase in cell output relative to input cells over time (Fig. 7C).

## Discussion

The ontogeny of L-DC development in perinatal spleen has been investigated with a view to determining the lineage relationship of these cells with other DC subsets. Phenotypic analysis of prenatal E18.5 spleen by antibody staining and flow cytometry revealed the presence of L-DC and other myeloid cells, but not cDC and pDC. Following birth, L-DC were still present, but pDC appeared later at 2 days, and cDC only at ∼4 days following birth. This time course clearly distinguishes L-DC developmentally from pDC and cDC.

E18.5 spleen cells showed production of only L-DC when co-cultured with 5G3 splenic stroma. This result is consistent with the presence of L-DC progenitors in prenatal spleen, and raises the possibility that L-DC progenitors are laid down during embryogenesis reflective of yolk sac-derived HSC and a separate lineage of DC. This is also consistent with reported higher frequencies of HSC in neonatal spleen [7], [29]. A model for a separate macrophage lineage arising from primitive HSC has been developed for tissue macrophages including red pulp macrophages in spleen [30], although our evidence to date, distinguishes L-DC from those cells.

In contrast to L-DC, cDC and pDC populations in spleen derive from described common dendritic progenitors (CDP) [27], [31] or myeloid dendritic progenitors (MDP) [32] in BM, which lead to DC precursors (pre-DC) which enter spleen via blood where they mature further [22]. The later development of pDC and cDC in the perinatal period could be attributed to their development via pre-DC arising from progenitors resident in BM. Pre-cDC form in BM and continually migrate to spleen via blood to serve as a reservoir for splenic cDC development and turnover [22]. In a similar scenario, tissue-resident monocyte populations derived from yolk sac progenitors have been defined [33] which are distinct from blood monocytes/macrophages, the latter arising from precursors in BM in the adult, entering blood and tissues as monocytes under inflammation [34]. In the case of L-DC, and also for residential monocytes, it would appear that hematopoiesis can occur from endogenous progenitors in spleen.

While there is increasing evidence that spleen contains HSC even in the steady-state [1], [3], [4], numbers in the adult are very low. We have shown this in previous tracing experiments where spleen cells of both neonatal and adult mice were shown to give broad lineage reconstitution of myeloid and lymphoid cells following adoptive transfer into lethally irradiated host mice [36], [37]. While all subsets of DC were found to develop, L-DC did however show higher representation amongst DC and myeloid cells developing in spleen suggesting that their development was spleen-specific. Our previous evidence also shows a distinct phenotype for HSC in 8 day old mouse spleen, being Lin^−^c-kit^lo^Sca-1^−^ cells [35], [36]. Flow cytometric analysis of one day old spleen here has shown the presence of both LT-HSC and MPP, having phenotypes more similar to subsets in BM [24], [28]. Both LT-HSC and MPP sorted from one-day old spleen showed restricted differentiative capacity for L-DC in stromal co-cultures with no evidence for production of cDC-like cells. At this stage, it is proposed that HSC/MPP in D1 spleen are endogenous to spleen, probably as yolk sac-derived HSC. Co-culture studies have revealed the direct and restricted differentiation of L-DC supported by 5G3 stroma which maintains hematopoiesis for at least 4 weeks, with maintenance of progenitor cells for that entire period. Evidence that both of the phenotypically distinct LT-HSC and MPP subsets contain L-DC progenitors can be explained by either the presence of two distinct progenitors for L-DC, or for the two progenitors being developmentally linked, such that one is the precursor of the other. This hypothesis has been proposed previously in a separate study of BM-derived L-DC progenitors [18].

Further evidence for lineage distinction between L-DC and cDC is that M-CSF is essential for cDC-like cell development, but redundant for L-DC development. This is consistent with different pathways for development, and probably the existence of distinct progenitors. All of these findings are consistent with evidence that L-DC but not cDC progenitors are present amongst LT-HSC sorted from BM as described previously [17], [18], or from D1 spleen as shown here. Whether splenic LT-HSC actually differentiate to give L-DC progenitors or even CDP in the splenic microenvironment, is still an open question.

The production of L-DC in cultures of perinatal spleen supplemented with Flt3L or GM-CSF/IL-4 was also investigated in relation to production of other DC like cDC. Indeed, Flt3L and GM-CSF/IL-4 have been used in many labs to grow out populations of DC from BM cells *in vitro*. DC developing in GM-CSF/IL-4 have been described as CD11c^+^MHC-II^+^ DC, and used for the study of immune responses in both mice and humans for over a decade [38], [39]. Flt3L addition to BM cells has been shown to induce the production of both cDC and pDC [27], [40]. Recently, Xu et al [26] compared DC production in BM due to Flt3L and GM-CSF and showed that Flt3L induced cDC and pDC, while GM-CSF induced monocyte-derived inflammatory DC [26]. When neonatal or adult spleen cells were cultured with Flt3L and GM-CSF/IL-4, they induced a range of cells expressing CD11c and MHC-II. The capacity of Flt3L to maintain myeloid DC (CD11b^+^CD11c^+^) was lower than that of GM-CSF/IL-4, suggesting that GM-CSF/IL-4 may be superior in DC expansion from existing precursors. However, since CD11b^+^CD11c^+^MHC-II^+^ cells were produced here from spleen cells in Flt3L supplemented cultures, a high number of Flt3^+^ DC precursors must be present amongst spleen cells. GM-CSF/IL-4 also induced a significant proportion of CD11b^+^CD11c^+^MHC-II^−^ cells in cultures which do not appear to be L-DC in that they are F4/80^−^Ly6C^+^ cells and so may reflect monocytic cells rather than L-DC (Figure 4B). F4/80 was recently shown to be a marker for L-DC *in vivo* and *in vitro* (Fig. 6; unpublished data). Flt3L supplemented cultures did produce a subset of CD11b^+^CD11c^+^MHC-II^−^F4/80^+^ cells. It would appear therefore that Flt3L can support L-DC production, as well as the production of cDC and monocyte-like cells.

Overall, the emergence of L-DC in prenatal spleen prior to the appearance of pDC and cDC, and their lack of M-CSF dependency, distinguishes them as antigen presenting cells in spleen. The possibility that they derive from spleen-endogenous self-renewing HSC and MPP is supported by evidence that LT-HSC and MPP subsets from D1 spleen also contain L-DC progenitors. Indeed, the presence of the L-DC subset in embryonic and newborn spleen supports a model for tissue-specific dendritopoiesis occurring in spleen. This is a new phenomenom which would support a model for compartmentalisation of the immune response with tissue-specific antigen presenting cells.
